# Chronic gastrointestinal bleeding caused by a Dieulafoy’s lesion in the small intestine: a case report

**DOI:** 10.1186/s13256-021-03121-9

**Published:** 2021-11-02

**Authors:** Javad Salimi, Mohamad Behzadi, Alireza Ramandi, Mehdi Jafarinia, Hamid Zand, Mohammad Pirouzian

**Affiliations:** 1 Vascular Surgery Department, Sina Hospital, Tehran University of Medical Sciences, Tehran, Iran; 2 School of Medicine, Tehran University of Medical Sciences, Tehran, Iran; 3 Vascular Surgery Department, Naft Hospital, Tehran, Iran

**Keywords:** Dieulafoy’s lesion, Gastrointestinal bleeding, Surgery, Small bowel, Case report

## Abstract

**Introduction:**

Dieulafoy’s lesion, first found by Paul Georges Dieulafoy, is an infrequent but important cause of recurrent upper gastrointestinal bleeding. The bleeding is usually severe, but patients rarely present with chronic, occult gastrointestinal bleeding.

**Case presentation:**

In this article, we discuss the case of a 68-year-old caucasian man with a history of recurrent hematemesis and chronic anemia with evidence of extravasation of contrast in the lumen of the bowel loop on computed tomography angiography. The patient was taken to the operating room, and a laparotomy procedure was performed.

**Conclusion:**

Due to the infrequency of Dieulafoy’s lesion compared with other causes of gastrointestinal bleeding, it is often missed in the process of differential diagnosis. In this article, we have demonstrated the importance of this disease and different approaches to the treatment of this lesion, considering the location of the lesion among other factors.

## Introduction

Dieulafoy’s lesions (DLs), first found by French surgeon Paul Georges Dieulafoy (1839–1911), were described as a superficial ulcer accompanied by a huge arteriole in the submucosal layer observed in the autopsied specimen. He described a series of ten patients presenting with massive hematemesis from a bleeding gastric vessel. However, they did not have any evidence of ulceration [1]. DLs account for 0.3–7.0% of nonvariceal upper gastrointestinal (GI) hemorrhages [2]. Macroscopically, this arteriovenous malformation (AVM) comprises a small-sized lesion appearing as a mucosal defect with an artery protruding from its base [3]. DLs are typically found in the lesser curve of the stomach (within 6 cm of the gastroesophageal junction) [4]. Massive bleeding from these lesions can be fatal unless adequate treatment is promptly initiated [5]. Patients are typically asymptomatic before presenting with acute, profuse GI bleeding, hematemesis, melena, or hematochezia. However, chronic, occult GI bleeding is an uncommon presentation in patients. DLs in the small bowel are very rare and are most frequently seen in the jejunum [7]. In this case presentation, we are reporting a DL located in the small bowel, diagnosis, management, and the follow-up required.

## Patient information

A 68-year-old Iranian man was investigated for about 12 months with a history of recurrent hematemesis and chronic anemia without any diagnosis. Due to multiple normal endoscopy and colonoscopy results, he was referred to us in the surgery department for further evaluation and care. Before the occurrence of bleeding, the patient was asymptomatic and without any significant complaints. Due to the intermittent nature of the bleeding, the patient had a normal fecal digital rectal examination without any signs of blood at the time of admission. According to the patient’s records during the bleeding periods, anemia and a significant decrease in hemoglobin (Hb, 8.7 g/dL) were found. He had no past medical history of any other diseases and mentioned a normal family and psychosocial history.

## Diagnostic assessment

**Fig. 1:**
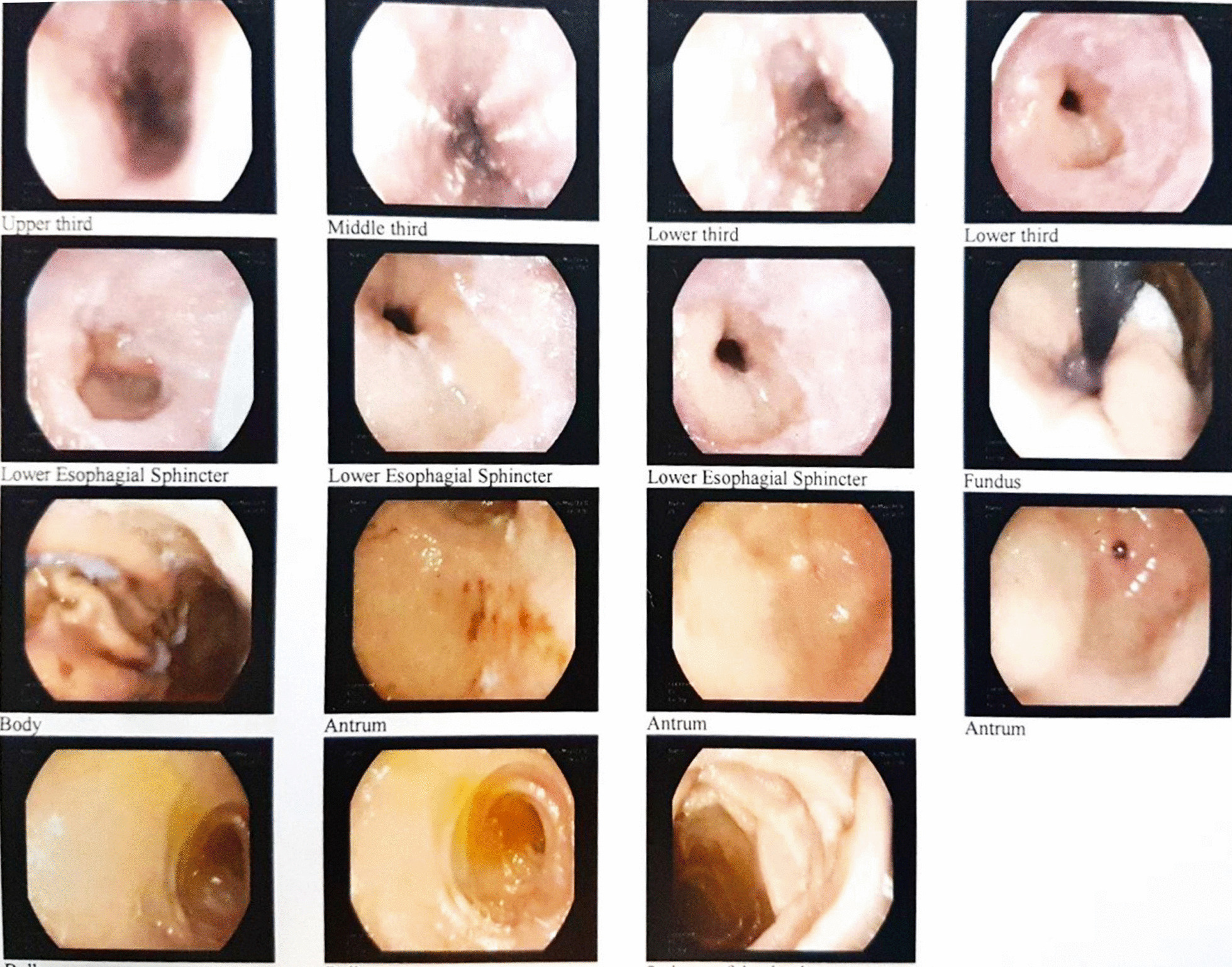
In the mucosa of middle lower third of esophagus, candidiasis along with diffuse extent esophagitis in lower esophageal sphincter; single papule in greater curvature of antrum of stomach. Duodenum was normal

**Fig. 2:**
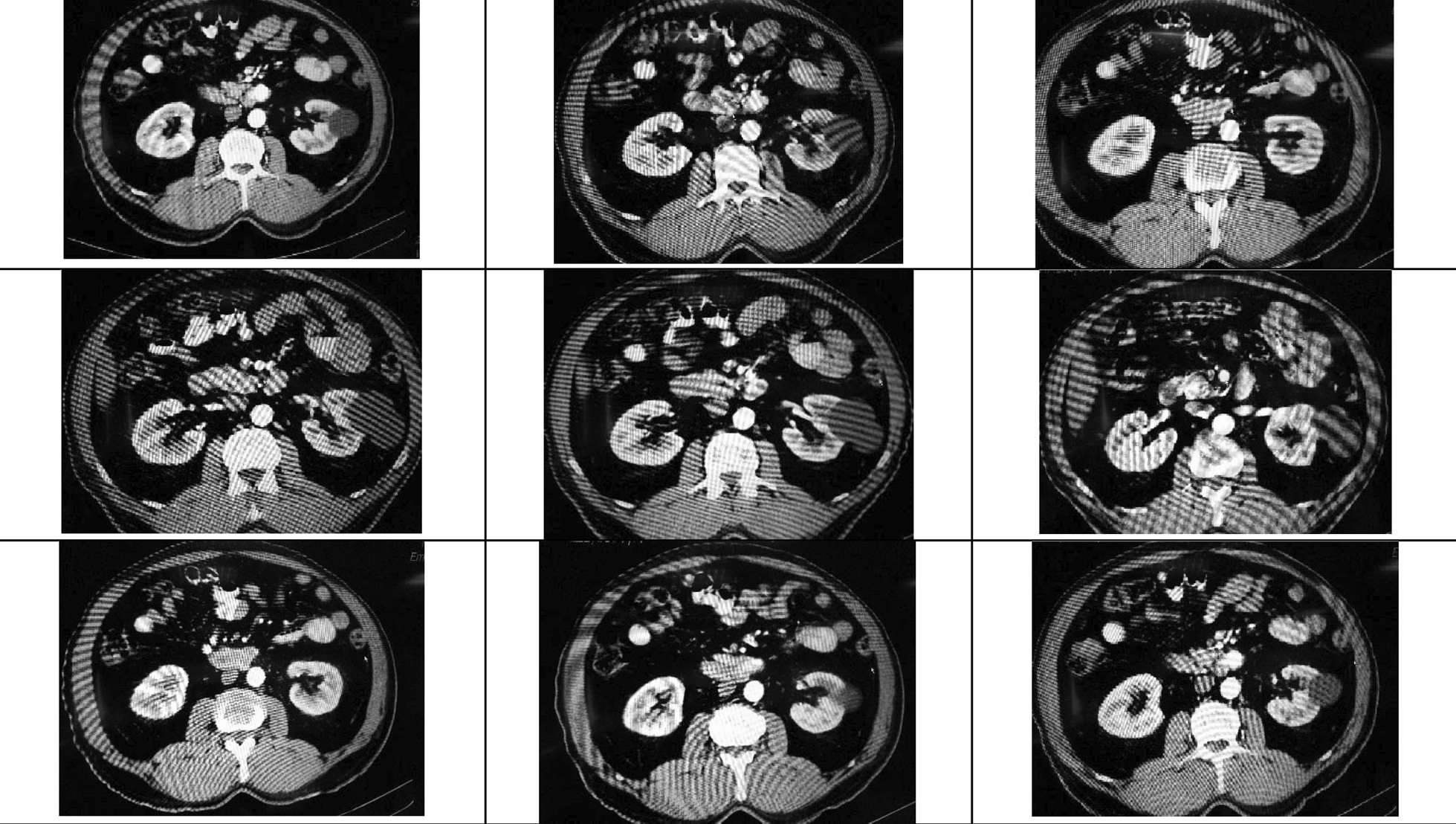
Multi-slice axial and reconstructed coronal and sagittal maximum intensity projection (MIP) images of the abdominal aorta revealing arteriovenous fistula in the territory of the jejunal branches of the superior mesenteric artery (SMA) with a saccular pseudoaneurysm protruding into the mid-jejunal loops. There is evidence of extravasation of contrast into lumen of bowel loop and early venous filling/arteriovenous (AV) shunt into branches

The physical examinations were normal between the periods of bleeding. During the long-term diagnostic approach, esophagogastroduodenoscopy was performed and observations included esophageal candidiasis, single papule in the greater curvature, and normal duodenum (Fig. 1). On colonoscopy, grade B hemorrhoid veins with rectal bleeding were observed, along with small opening diverticula in the sigmoid and descending colon, hence not accounting for all the signs and symptoms observed. Since none of our findings revealed the source of the bleeding, further diagnostic workups were necessary. We found the region of the bleeding through computed tomography angiography (CTA). On CTA, there was evidence of extravasation of contrast in the lumen of the bowel loop (Fig. 2).

## Intervention and follow-up

**Fig. 3:**
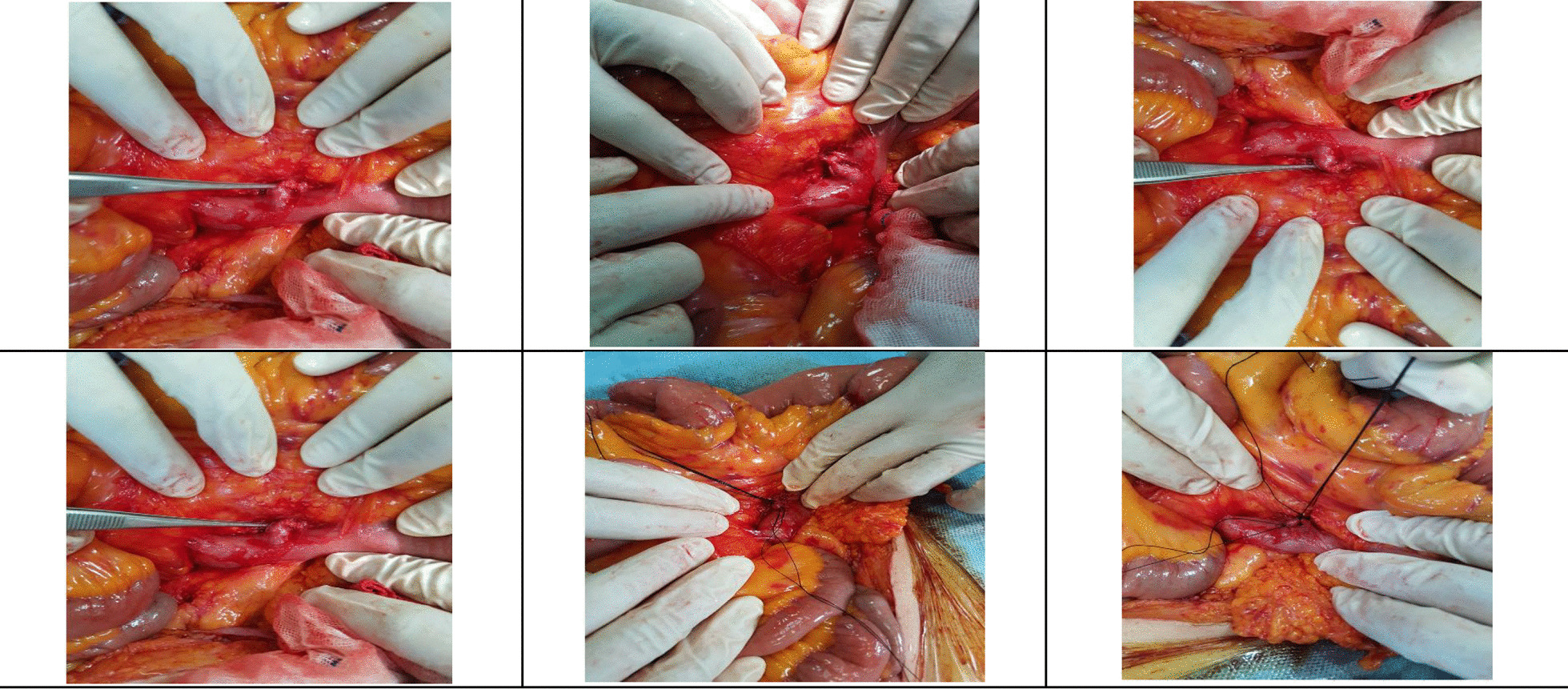
Arterial resection followed by vascular anastomosis performed suture threads and the forceps are pointing to the adjacency of the artery to the serosal lining of the small intestine, indicative of a Dieulafoy lesion

The approximate location of the lesion was estimated from the CTA results. A surgical approach was chosen as soon as the region of bleeding was found. The patient was taken to the operating room, and a laparotomy was performed under general anesthesia. We searched for the DL in the small intestine mesentery. After observing the DL, arterial resection was performed, followed by vascular anastomosis. The small intestine was intact, and no further intervention was necessary (Fig. 3). During the follow-up, CTA was routinely performed as a part of the practice 36 months after the operation, in which there were no signs of any aneurysms, stenosis, dissection, or mural thrombosis in the abdominal aorta. Hence, no evidence of recurrence was found on follow-up.

## Discussion

DL is an infrequent but important cause of recurrent upper gastrointestinal bleeding. The size of the mucosal lesions is most usually between 2 and 5 cm [3]. These lesions account for 0.3–7.0% of upper gastrointestinal bleedings and could be found in 1–2% of patients who have undergone upper gastrointestinal hemorrhage surgery [2, 3]. Chaer *et al.* suggested that the prevalence of these lesions should be described as “unrecognized” rather than rare [3]. Although DL could be found anywhere throughout the gastrointestinal tract and in the bronchus, the most frequent region is on the lesser curve of the stomach [4]. DLs in the small bowel are uncommon. However, the hemorrhage resulting from DL in the small bowel could be life-threatening. Patients with DL in the small bowel would rarely present with chronic occult gastrointestinal bleeding, making it an area of interest in our evaluations.

The diagnosis of DL was previously achieved upon classical histologic features after the procedures of laparotomy exploration, autopsy, or the surgery performed [8]. However, histologic evaluation is usually unavailable. Small bowel bleeding continues to be challenging to visualize directly on routine endoscopy. Therefore, the diagnosis of bleeding in the small bowel is often delayed [9]. To localize the region and the source of small bowel bleeding, multidisciplinary approaches such as abdominal CT, angiography, radionuclide scan, and capsule endoscopy are needed. In this case, a variety of investigations had been performed, but the small bowel lesion is difficult to visualize, and eventually, after CTA we found the region of the bleeding. On the CTA, there was evidence of extravasation of contrast in the lumen of the bowel loop.

Patients with DL usually present with hematemesis associated with melena [3]. Hence, the patients should first be treated using a general GI bleeding approach, necessary fluid replacement, blood transfusions, and blood products. Close follow-up monitoring should also be provided. The principles of management of DLs depend on their location [10]. Endoscopic therapy achieves permanent hemostasis in 85% of cases in gastric, duodenal, and proximal jejunal lesions, with only 5% of cases proceeding to surgery [8]. In the DLs located in proximal part of small bowel, surgical exploration with intraoperative endoscopy has yielded excellent results and avoids resection. Segmental resection and anastomosis is the preferable choice to treat small bowel DL.

## Conclusion

Dieulafoy’s lesions (DLs) could be life-threatening, acute, or chronic. Surgical treatment is advantageous with a low risk of re-bleeding, and surgery should be selected when patients are hemodynamically unstable and when other methods have failed. In conclusion, we recommend that DL should be considered as an important differential diagnosis in acute and chronic GI bleeding. Although endoscopic modalities are chosen to treat DL, segmental resection and anastomosis are preferred in small bowel DL treatment.

## Data Availability

Not applicable.

## Abbreviations

AVM: Arteriovenous malformation
CTA: Computed tomography angiography
DL: Dieulafoy’s lesion
GI: Gastrointestinal
